# The perception of health care quality by primary health care managers in Ukraine

**DOI:** 10.1186/s12913-022-08300-y

**Published:** 2022-07-10

**Authors:** Valentyna Anufriyeva, Milena Pavlova, Tetiana Stepurko, Wim Groot

**Affiliations:** 1 Department of Health Services Research, CAPHRI, Maastricht University Medical Center, Faculty of Health, Medicine and Life Sciences, Maastricht University, Maastricht, The Netherlands; 2 Ukrainian-Swiss Project “Medical Education Development”, Swiss Tropical and Public Health Institute, Kyiv, Ukraine; 3 Department of Sociology, National University of Kyiv-Mohyla Academy, Kyiv, Ukraine; 4 Top Institute Evidence-Based Education Research (TIER), Maastricht University, Maastricht, The Netherlands

**Keywords:** Health care quality, Perceived quality, Service quality, Health care management, Primary health care, Ukraine

## Abstract

**Background:**

Ukraine is reforming its health care system to improve quality of health care. Insight into how primary health care managers perceive quality is important for the ongoing reform as well as for the improvement of medical services.

**Methods:**

An online survey was conducted as part of the Ukrainian-Swiss project “Medical Educational Development” in April–May 2019 based on the contact list of USAID project “Health Reform Support”, and additionally on the database of the National Health Service of Ukraine and other channels. Data were analyzed using descriptive statistics and qualitative data analysis.

**Results:**

In total, 302 health care managers took part in the study. The majority of primary health care managers perceive quality in health care as process quality. They associate quality mostly with compliance to standards. At the same time, primary health care managers prefer to assess outcome quality via a system of indicators and feedback. There appears to be a lack of consensus about health care quality. This may be due to a lack of awareness of the national strategy for better quality of health care service.

**Conclusions:**

Our study provides new insights into primary care managers' perceptions of health care quality in Ukraine. The absence of a clear consensus about quality complicates the discussion about quality and how to measure quality in health care. This appears to be one of the obstacles to system-wide quality improvement.

**Supplementary Information:**

The online version contains supplementary material available at 10.1186/s12913-022-08300-y.

## Background

In 2015, Ukraine initiated a reform of its health care financing. The aim of the reform was to improve health and to lower the financial burden on the population. Initially, the reform focused on primary health care; reforms of secondary and tertiary health care followed in 2020. The financing of the primary care system was changed from hospital-oriented fixed line-item budgeting to per-capita financing. Primary health care providers became more autonomous [1]. This also included a change in management. Traditionally only physicians or nurses could be appointed as health care managers (e.g., head doctor, head nurse, etc.). Since 2020, health care managers with managerial and non-medical background are also allowed to take managerial positions [2]. The reform is supposed to result in a more modern, competitive and high-quality system of medical care [3].

What is still absent from the system is a national policy on health care quality and a national quality strategy for health care. Health care quality is defined in the Order of the Ministry of Health #752 dated September 28, 2012 as follows: “providing medical assistance and organizing health care services according to health care standards. Health care quality assessment is the compliance of medical assistance provided to formalized health care standards”. At the same time, health care standards are not defined in Ukraine [1].

The classic Donabedian’s quality model describes quality by three system elements: structure, process and outcome [4]. Structure refers to the resources, personnel, administration and facilities. Process includes performance management, patient records, diagnosis, treatment plan. And outcome includes patient satisfaction, health status, completion of treatment, and recall pattern [5]. This model of health care quality is widely used.

In practice, however, the perception of health care quality varies depending on the context and perspective of the different stakeholders [6]. In particular, different stakeholders use different indicators, so-called quality attributes, to define and assess quality. Health care professionals tend to perceive quality through the concordance of clinical results with guidelines [6]; work environment and job satisfaction [7]; physician leadership, infrastructural support, culture of the organization and valid health care quality measurement and evaluation [8]; clinical governance and leadership [9]. For patients, quality depends on good care and treatment, health improvements, a clean and homelike service environment and interactions with the service provider [6]. Policy makers often believe quality indicators like accessibility, equitability and satisfaction of both health care users and providers to be important [6]. The perception of quality among stakeholders in general and health care managers, in particular, is important because it influences the implementation of reforms [10] at a system level as well as the choice of tools for quality management at the level of a facility.

Several studies on health care professionals’ perceptions of quality of health care have been conducted [9, 11–15]. Some of these studies have focused on primary health care [16–22]. These studies identify organizational aspects that affect the quality of interventions, physicians’ performance, team performance and health care system effectiveness. Among the quality attributes, health care professionals point at the general practitioner’s (GP) role [16, 17], positive work attitudes [18], physicians’ mental health [16], nurses’ competencies [19], organizational quality orientation [18], accessibility [20] and clinical leadership [18] as indicators of quality. Whereas patients find doctor-patient relationships [21], organization of care [21], access to care and adequacy of waiting times to be important [19]. Studies have also compared patient and physicians’ assessments of quality and have concluded that perceptions differ between groups and are often based on a different logic, e.g., physicians are more critical about quality than patients and tend to underestimate the level of positive attitude of their patients [21].

There are only a few studies on quality of health care in Ukraine [10, 23]. In particular, Peabody et al., 2014 studied quality of health care services in Ukraine in 2009 and 2010. In that study, quality of clinical care for congestive heart failure and chronic obstructive pulmonary disease was assessed through a vignette analysis of clinical quality. Quantitative data obtained from medical facilities, physicians, patients at the facilities and households showed no significant differences in quality between urban and rural medical facilities, or between facilities of different levels. Quality also did not vary significantly if a physician worked in several facilities or had a higher number of elderly patients. Another study reported on the perspectives on quality and on the effectiveness of the health system in Ukraine [23]. The data were collected in 2009 and 2010 among household representatives (adults), physicians and clinic patients. The participants described quality through physician training, the amount of time spent with patients, and accessibility and affordability of care. The results showed that the health care system reforms and the improvement of quality and affordability should become the major goals of the new policies [23]. The quality indicators of both studies, however, measure process quality and not outcome quality.

We did not find studies on the perception of quality of health care managers in Ukraine and in particular, no such studies have been performed after the launch of the reform. It is therefore essential to study how health care managers in Ukraine perceive quality because understanding their focus is important for the reforms and to achieve consensus about the objectives in health care [10]. Many countries with similar healthcare systems undergoing similar changes lack evidence on the impact of the reforms on their healthcare system [24]. Thus, evidence on Ukraine’s experience might also be useful for countries with similar health care systems in transition.

The aim of this study is to describe the perception of quality by primary health care managers in Ukraine. We expect to identify the quality attributes identified by the health care managers as descriptors of quality and ways of quality assessment used in the everyday practice of Ukrainian health care facilities. As we will show, the perception of quality among primary health care managers differs widely and includes statements like ‘one of the ten categories defined by Aristotle’ and ‘something unreachable for rural medicine’.

## Methods

We used data from the online survey “Educational opportunities for managers in health care of Ukraine” conducted in April–May 2019 by the Ukrainian-Swiss Project “Medical Education Development” (MED). The aim of the survey was to identify the educational needs of primary health care managers and their expectations towards the form and content of lifelong learning.

An online survey was used as the data collection mode. Online surveys are suitable for gathering information about health care professionals’ attitudes and opinions. Among the main advantages of this mode there are the possibility of tailoring to the situation, low response bias and low cost as well as flexibility for the participants who are usually pressed for time and are difficult to reach via telephone or face-to-face. Health professionals appear to be 10–13% less likely to participate in surveys than the general population and the rate of participation is constantly decreasing because, among other reasons, they usually have to do it in their personal time, often consider it as irrelevant, suffer from information overload and privacy concerns [25].

The questionnaire contained four blocks of both open-ended and closed-ended questions. In particular, the block “Quality management” contained two open-ended and one closed-ended question to clarify the understanding of the notion “quality”, and whether there is a quality management system in the facility and how quality is assessed in the health care facility (see Additional file 1:Appendix A).

Thus, three questions concerning health care quality management were used to collect data on the perception of health care quality by primary health care managers in Ukraine and the way quality is measured at their health care facilities: “What does the term ‘quality in health care mean to you?”, “Do you have a quality management system in your health care facility?”, and “If you have a quality management system in your health care facility, please, describe how you assess quality.” This study focuses on the data gathered through these three survey questions.

Prior to the survey, the questionnaire was validated by experts who read and commented upon it as well as pre-tested. Five health care managers (head doctors of primary facilities) were asked to fill in the questionnaire and comment on the questions. The questionnaire was modified based on these comments but the wording of questions mentioned above stayed the same as no suggestions for change were made.

The sampling units were health care managers (chief doctor, deputy chief, head of department, chief nurse) as well as those who were on the “reserve list” for a management position at a primary health care facility.

A mixture of sampling methods was used. First, a link to the online survey along with a request to participate was sent to health care professionals in the contact list of the USAID project “Health Reform Support”. This list contained the contact information of primary health care managers who took part in USAID projects. The risk of bias in the sample selection is a known disadvantage of this method. Our sample also contains a small number of respondents who are not managers.

As the participation rate was low, a total population sampling method was used: a link to the online survey along with the request to participate was sent to health care professionals via the database of the National Health Service of Ukraine (NHSU). The database contained contact information of all primary health care managers who worked with the NHSU.

In addition, the survey link, along with the invitation to participate, was posted on the Facebook page of the MED project (a convenience sampling method). Two reminder e-mails were sent to health care professionals in the contact list of USAID and the NHSU at ten-day intervals. After that, on the eleventh day after the second reminder e-mail, the online survey form was closed.

As we do not have access to the contact lists of the NHSU or the USAID project, it is impossible to determine the response rate.

The answers to the open questions were first coded and then analyzed using descriptive statistics. Regarding the open-ended questions “What does the term ‘quality in health care’ mean to you”, the responses were given in two ways: enumerating keywords associated with quality or giving a complete sentence. We grouped the answers to this question into three major groups following the Donabedian’s quality model: quality of structure, quality of process and quality of outcome. One answer could be classified into more than one group. Various attributes and tools were identified in each group based on the participants’ understanding of quality. Responses related to the following attributes were classified as quality of structure: integration, efficiency, organization and administration (management), and qualification. Attributes included in the quality of process were: effectiveness, people-centeredness, safety, timeliness, equity, service and compliance to standards. The quality of outcome included responses related to the following attributes: indicators, such as the morbidity rate, health index, mortality rate, number of treated cases, vaccination rate, etc., absence of complaints, patient satisfaction, and doctor satisfaction.

We also analyzed the responses according to their similarity to three definitions of quality most frequently used in Ukraine:

- The definition of the Institute of Medicine, which includes structure, process and outcome and focuses on effectiveness, safety, people-centeredness, timeliness, equity, integration, and efficiency [26].
- The definition of the European Commission with its focus on effectiveness, safety and people-centeredness – the attributes of the process [27].
- The Ukrainian definition with its focus on attribute of the process quality—compliance to standards [28].

Descriptive statistics and qualitative data analysis were also used to analyze the responses to the other two questions: “Do you have a quality management system in your health care facility?”, and “If you have quality management system in your health care facility, please, describe how you assess quality.”

Qualitative data analysis was performed following the “bottom up” approach [29]. The data was first sorted into themes. The results of the sorting as well as the discrepancies were discussed by all authors. The data were then coded by means of assigning short phrases to each response. The results of coding as well as the discrepancies were also discussed by the authors. The results of our study are confirmed by another small-scale study.

Ethical considerations. All participants were fully informed about the purpose of the study, how the findings would be used, whether there were any adverse impacts of their participation and who would have access to the findings. This information was presented in the cover e-mail, which introduced the questionnaire as well as in the opening statement of the online questionnaire. At the beginning of the questionnaire, the participants ticked the box (informed consent) to confirm that they were fully aware of the purpose of the study and further usage of the data. Participants were also reminded that they were free to withdraw their participation at any time without any negative impact. No identifying information was made available to any other parties. Ethical approval was not obtained as, according to the Ukrainian regulation, it is not necessary for research of this kind.

## Results

In total, 354 online questionnaires were filled in by health care professionals. Twenty eight participants worked at secondary level hospitals and five at academic hospitals. In this study we focus on primary health care managers and exclude from the further analysis these thirty three hospital managers. Nineteen participants completed the survey twice. For these participants, the first filled-in questionnaire was included. After the duplicates were removed, 302 completed questionnaires were used in the analysis. Out of these, 19 questionnaires had no personal data information (name, gender). One questionnaire contained answers only for the close-ended questions. Two questionnaires contained no answers to the question “What does the term ‘quality in health care’ mean to you?”, and three questionnaires had no answer to the question “If you have a quality management system in your health care facility, please, describe how you assess quality” even though the participants indicated that a quality management system was present. However, no questionnaire was excluded from the analysis because of missing data.

Table 1 contains information about the participants and the health care facilities they work at. The majority of the participants (67.9%) were female. The majority of the respondents (50.7%) were more than 45 years old.

**Table 1:** Participant characteristics

Characteristics	Number % *n* = 302
Gender -Male -Female -Not reported	78 (25.8%) 205 (67.9%) 19 (6.3%)
Age -25–35 -36–45 -More than 45 -Not reported	60 (19.9%) 88 (29.1%) 153 (50.7%) 1 (0.3%)
Position -Director -Deputy director -Chief doctor -Deputy chief doctor -Chief of the department -Doctor -Nurse – administrator -Other	60 (19.9%) 17 (5.6%) 117 (38.7%) 39 (12.9%) 18 (6%) 33 (10.9%) 2 (0.6%) 16 (5.3%)
General experience (years) -0 -1–5 -6–10 -11–20 -More than 20 -Not reported	0 20 (6.6%) 28 (9.3%) 88 (29.1%) 165 (54.6%) 1 (0.3%)
Managerial experience (years) -0 -1–5 -6–10 -11–20 -More than 20 -Not reported	41 (13.6%) 101 (33.4%) 73 (24.2%) 66 (21.8%) 19 (6.3%) 2 (0.6%)

The majority of health care professionals held managerial positions. The category “doctors” (10.9%) included private practitioners (5 out of 33), medical doctors from the reserve list waiting to be appointed on a managerial position (5 out of 33). The category “others” (5.3%) included a specialist in communications, an economist, a legal adviser and a human resources officer.

In general, participants were very experienced in clinical work but had much less managerial experience.

As described in Table 2, in 4.3% of the cases, the participants’ answers regarding the definition of quality could be related to the Institute of Medicine definition with its focus on structure, process and outcome quality. In 30.8%, the answer referred to the Ukrainian definition with its focus on process and in 40.4% to a combination of definitions. Quality aspects defined by the European Commission were only found in combination with the other two definitions in 24.1% of the cases. Elements of the Ukrainian and the Institute of Medicine definitions were most frequently combined (34% and 32.6%, respectively).

**Table 2:** Quality perceptions

Characteristics	Number % *n* = 302
Answer characteristics -Own words (comprehensive) -Key words -I do not know -Missing answer	202 (66.9%) 97 (32.1%) 1 (0.3%) 2 (0.6%)
Compliance of the answer to^a^ -Institute of medicine term (IM)^b^ -Ukrainian term (ukr)^c^ -European Commission term (EC)^d^ -Combination of terms -Other	13 (4.3%) 93 (30.8%) 0 122 (40.4%) 71 (23.5%)
Combination of terms^e^ -Ukr + IM + EC + other -Ukr + IM + EC -Ukr + IM + other -IM + EC + other -Ukr + IM -Ukr + other -IM + EC -IM + other	19 (6.3%) 40 (13.2%) 8 (2.6%) 4 (1.3%) 13 (4.3%) 23 (7.6%) 10 (3.3%) 5 (1.7%)
Quality aspects mentioned -Structure -Process -Outcome -Other	94 (31.1%) 294 (97.3%) 126 (41.7%) 18 (5.9%)
Number of aspects mentioned per one answer ^a^ -1 -2 -3 -4 -5 -6 -7	172 (56.9%) 64 (21.2%) 29 (9.6%) 16 (5.3%) 13 (4.3%) 1 (0.3%) 3 (0.9%)

*Note*: ^a^One answer could not be complied with any of the terms under analysis and contained no mention of aspects. “Quality is something unreachable for rural medicine”

^b^“The degree to which health services for individuals and populations increase the likelihood of desired health outcomes and are consistent with current professional knowledge” [26]

^c^“Providing medical help and organizing health care services according to the health care standards. Health care quality assessment is the compliance of provided medical help to the formalized health care standards” [28]

^d^“Health care that is effective, safe and responds to the needs and preference of patients” [27]

^e^Combination of terms comprised 40.4% of the general number of answers

Table 2 also shows that in most cases (66.9%), the answers were in the form of complete sentences. And in 32.1% of the cases, the participants described quality enumerating keywords. There were two missing answers (0.6%) and one “I don’t know” answer (0.3%).

The majority of the answers (97.3%) interpreted quality in health care as process quality. Structure and outcome quality attributes were mentioned in 31.1% and 41.7% of the answers, respectively. A group of answers (5.9%) that did not describe any process, structure or outcome quality attributes was included in the category “other”. This group included quality attributes such as basic social rights, the creation of a medical services market, reforms, etc.

In 56.9% of the cases, one attribute was mentioned in the response. In the rest of the cases, quality was associated with two or more attributes.

**Fig. 1:**
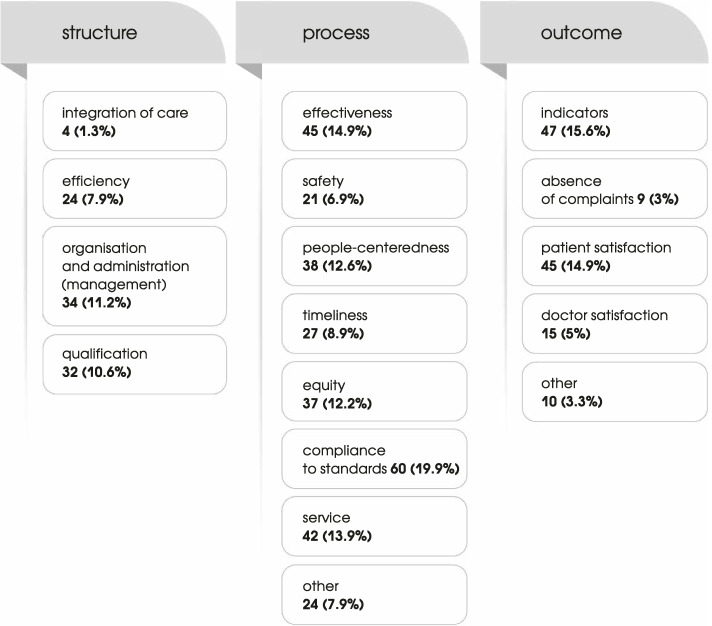
Aspects of quality in health care as understood by participants. Percentages indicate the share of participants who indicated the given attribute. The answers like "team work", "preciseness" and "qualitative medical help" were calculated as "process aspects mentioned" (7.9%) and are shown as "other". The answers like "satisfaction" without spcifying patient or doctor, or "quality of life" were calculated as "outcome aspects mentioned (3.3%) and are shown as "other"

Using the model shown in Fig. 1, we describe with what attributes primary health care managers in Ukraine associate quality in health care and how frequently each attribute was mentioned. Tables 3, 4, and 5 present quotations of health care managers describing quality attributes.

As can be seen from the tables, health care managers in Ukraine mostly associate structure quality with management (11.2%) and least of all with integration of care (1.3%). Process quality is strongly associated with compliance to standards (19.9%) and effectiveness (14.9%). It is less strongly associated with timeliness (8.9%) and safety (6.9%). Outcome quality is described by health care managers through indicators (15.6%) such as the morbidity rate, health index, mortality rate, number of treated cases, vaccination rate, etc. Outcome quality is also associated with patient satisfaction (14.9%) and doctor satisfaction (5%).

In general, the most frequently mentioned attributes of quality were compliance with standards and indicators, whereas the least mentioned was the integration of care. Regarding the “medical service” attribute, the answers did not allow us to determine what understanding of “service” health care managers in Ukraine have.

As shown in Table 3, structure quality is mostly perceived as “getting the best results quickly and without unnecessary spending”. Quality is associated with modern equipment, correct organization of work and high professionalism of the medical employees.

**Table 3:** Quality aspects mentioned. Structure

Quality aspect	Quotation
Integration of care	“Integration between the medical facilities”
Efficiency	“Modern medical equipment” “Prevention and treatment without unnecessary expenses” “When the best results of treatment or diagnostics are received with the least resources” “Effective treatment for a shortest time period and with the minimum price”
Organization and administration (management)	“Combination of services and process management which bring the facility to the desired level of quality” “The main target function and criterion of the facility functioning” “Correct organization of employees work” “Control system”
Qualification (professionalism)	“These are professionally ready medical workers with constant development of their knowledge and responsible attitude to their work” “Responsible attitude. Qualified medical assistance to patients. Following ethics and deontology” “High level of specialized knowledge and skills, critical thinking and compliance to moral and ethical norms in treating patients”

Table 4 shows the perception of process quality. Health care managers mostly associate process quality with evidence-based treatment, comfortable conditions for patients, safety, patient needs, satisfaction and qualitative services in accordance with the standards.

**Table 4:** Quality aspects mentioned. Process

Quality aspect	Quotation
Effectiveness	“Evidence-based treatment, precise timely screenings and promotion of prevention” “Creating conditions for providing qualitative medical assistance to patients, patients’ comfort, timely diagnostics and treatment according to international and local protocols” “Receiving positive complex result in patient treatment on all the stages of medical service provision”
Safety	“Risk management” “Safe medical service” “Safety for patients” “Protection from possible risks and complications”
People-centeredness	“Fulfilment of justified patient needs” “Services that improve public health” “To fulfill patients’ needs in recovery and health maintenance”
Timeliness	“Timely primary medical assistance” “Speedy medical service” “Medical assistance for minimum time”
Equity	“Equality and equity” “To organize and support equity of medical services to public” “Access to all the sources of health care”
Compliance to standards	“Quality in health care is possible only in presence of standards” “Performance in accordance to international standards, approved and understandable actions for everyone” “Compliance to standards that support optimal conditions for services provision”
Service	“Opportunity to provide medical services on guaranteed high-quality level” “Perfect service in accordance to patient surveys” “Qualitative medical services to public”

As shown in Table 5, outcome quality is associated by health care managers with standardized indicators for different aspects of health related to prevention and treatment, patient satisfaction with services and doctor satisfaction with their job, labor conditions, and payment for work.

**Table 5:** Quality aspects mentioned. Outcome

Quality aspect	Quotation
Indicators	“Prevention actions coverage, reduce mortality, improvement of general health of the population” “Timely detection of oncological diseases and tuberculosis, reducing morbidity of heart diseases and other non-inflectional diseases” “Decrease of treatment length and number of days of work incapacity, decrease of number of chronic diseases aggravation” “Unification of standardized indicators for different levels of medical help” “Number of treated cases” “High vaccination coverage, healthy nutrition promotion” “Increase of number of citizens who signed declaration with the family doctor” “Performance indicators fulfillment actually and not statistically” “Percentage of valid diagnosis”
Absence of complaints	“Absence of complaints”
Patient satisfaction	“Patient is 100% satisfied with the service” “Satisfied healthy patient” “Patient assessment of the received medical help in a medical facility”
Doctor satisfaction	“Satisfaction of medical personnel with labor conditions” “Financial satisfaction of doctors” “Job satisfaction”

Table 6 presents the answers to the question “How do you assess quality in your health care facility”. This question was asked if a participant indicated that a quality management system was implemented in the health care facility, which was the case for half of the responses (52.6%). Structure quality was mentioned as the focus of quality assessment in 6.6% of the cases, process quality—in 12.2% of the cases, and outcome quality – in 21.5% of cases. At the same time, 26.8% of responses did not indicate the focus of the assessment.

**Table 6:** Quality assessment

Quality assessment characteristics	Number % *n* = 302
Quality management system is implemented in health care facility Yes No	159 (52.6%) 143 (47.3%)
Object for assessment^a^ Structure Process Outcome Not clear from the answer Missing answer	20 (6.6%) 37 (12.2%) 65 (21.5%) 81 (26.8%) 16 (5.2%)
Assessment tools used^a^ System of monitoring and evaluation Medical records assessment Feedback (satisfaction surveys both patient and medical staff, work with complaints) Expert meetings (morning conferences, treatment committees, pathological anatomical committees) Audits (both internal and external) Unclear from the answer Participant does not know Missing answer	104 (34.4%) 11 (3.6%) 32 (10.6%) 14 (4.6%) 8 (2.6%) 25 (8.3%) 1 (0.3%) 16 (5.3%)

*Note*: ^a^out of 53% those who had quality management system implemented in the health care facility. One answer could contain several objects for assessment and/or several assessment tools indication

As reported by the participants, the following tools were used to assess quality: a system of monitoring and evaluation (34.4%), medical records assessment (3.6%), feedback system, namely surveys, work with complaints (10.6%), expert meetings (4.6%), and audits (2.6%). In 8.3% of the cases, the response did not contain information on the exact assessment tools. Examples of such responses included: “self-control”, “we are still working at the system”, “in a way as I understood after asking my colleagues”. Two of these responses referred to legislative acts in Ukraine, one mentioned ISO certification and one mentioned the position of an employee responsible for quality in the facility. Several responses rated quality in their facility as “good”, “not enough”, “nine out of ten”.

## Discussion

This study investigated how primary health care managers in Ukraine perceive quality of health care. Without consensus and reliable information about quality, it is impossible to differentiate between adequate and poor quality of health care services. At the system level, the purpose of measuring quality lies in the need for external accountability and verification. Whereas on the local level, the focus is on quality improvement [27]. Thus, knowing the perceptions of quality by different stakeholders (e.g. health care managers) adds to our understanding of quality and is a first step to assess and improve quality. Also, managers’ understanding of quality influences patients and medical doctors as well.

Our study focused on perceptions of quality by primary health care managers in Ukraine. During medical training in Ukraine, quality and management are not addressed adequately (still, this aspect is underreported and understudied). Most of the health care managers have been trained as medical doctors and professional development for them is considered as clinical training. Health care managers get acquainted with definitions of quality and approaches to its assessment during their further education. After medical doctors are appointed as managers, they are expected to increase their skills in management within the framework of the Continuing Professional Development (CPD) programs. In Ukraine, medical doctors frequently do not speak or read English [30], which means that international sources are largely inapplicable. This limitation in language skills combined with outdated CPD programs [30] makes it difficult for health care managers to search for information to update their knowledge about quality, try different strategies and formulate a definition of quality of their own. To address the need of more up-to-date training for health care managers [30] the Ukrainian-Swiss project “Medical Education Development” developed online courses, among which there is also a “Quality Management in Health Care” course. More information about the course can be found at the MED project’s website [31].

The role of primary care lies in the management of the health of the population through a range of health care services like diagnostics, chronic illness management and further referral to medical specialists, drug prescription and health promotion [32]. All this makes primary care process-oriented. A limitation of a focus on process is that it ignores quality attributes like tangibles (structure) or patient satisfaction (outcome).

Ukraine has the long history of a top-down culture under which all the managerial decisions were taken by the Ministry of Health and the health care managers were to follow them. Also, the quality of Ukrainian health care providers is evaluated by an accreditation committee. Accreditation mainly focuses on procedures and is done by checking documental proofs of compliance to standards, constant professional development, etc. An accreditation certificate is valid for three years. As the study of the World Bank [33] on health care facility management of 2013 indicates, health care providers report to the State Medical Statistics Center of the Ministry of Health of Ukraine against the list of indicators. Between accreditations, other state bodies have the right to perform routine inspections of health care providers such as the Fire Inspection, the State Tax Service, the Social Insurance Fund, and the Ecological Control Service. At the same time, patient complaints are used for snap inspections and punishment actions by the dedicated government agencies. Because of this top-down organization, Ukrainian health care managers are not enthusiastic about structural reforms and the introduction of service quality control [33].

Health care facilities are traditionally closed communities. The informal “rules of the game” within the healthcare facility are created by the chief doctor. The interrelations between levels are not clear. Referral of a patient to another level or another facility depends on personal contacts of the doctor [33]. The problem here lies in the coping strategies of patient such as self-referral to specialists, out-of-pocket payments which result in a low utilization of primary health care services and a low level of trust.

Thus, our results indicate that a clear and uniform notion of quality is absent among primary health care managers in Ukraine. They tend to associate quality with one attribute only. The associations are, however, quite diverse as shown by our study. The primary health care managers in our study are mostly focused on process quality. The frequency of mentioning the “compliance to standards” and “indicators” attributes confirm the traditional focus of the Ukrainian approach to quality and show the lack of association of quality with integrated care.

A high number of unclear descriptions of measurement tools and answers like “quality is good/satisfactory” to the question on how quality is assessed, could have two major explanations. The participants did not distinguish between quality assessment (as a process) and the quality level in their facilities. Or the formulation of the question was unclear for the participants. Thus, the question of routine application of measurement tools in health care management practice requires further study.

The gaps in continuous professional development for managers, the lack of open dialogue and discussion on the priorities and challenges of service provision (in addition to the limited evidence available and published results on the perception of quality in health care) seem to be regional peculiarities. One recent study [34] describes the lack of horizontal exchanges, the almost absent learning culture to prevent mistakes in the neighboring countries to happen in Ukraine as well. However, we observe that there is attention to the perception of quality among health care managers, and health care professionals (and evidence that confirm the developed quality management systems) in other contexts [11, 22].

Our study has some limitations that need to be acknowledged. We focused on primary health care only, which left the understanding of quality in the Ukrainian hospital sector unexplored. The link used to distribute the online questionnaire was sent to potential participants by others. Thus, we had no access to the contact information, making it impossible to determine the response rate. A lack of generalizability is the known disadvantage of the convenience sampling method. We compare the results of our study with the results of a similar study to analyze the robustness of our results. In addition, we were unable to obtain details regarding the perception of quality among Ukrainian health care managers. For example, many participants (13.9%) named “service” as a quality attribute without providing an additional explanation. The perception of service by health care managers requires further research.

## Conclusions

In conclusion, our study provides new insights into primary care managers' perceptions of health care quality in Ukraine. Overall, our findings provide evidence for the existence of little consensus about quality among Ukrainian health care managers. We identified fifteen groups of quality attributes and still the meaning of some of them requires further clarification. Furthermore, most Ukrainian primary health care managers who took part in our survey do not recognize the multidimensionality of quality as more than half of the participants associate quality with one attribute only. This needs to be considered in future health care reforms.

Although some improvements have been made in health care financing reform, the health care system still lacks a national policy and dialog on quality and a national quality strategy for health care. The development and promotion of a national policy on quality and a national quality strategy for health care should become one of the priorities of the health care sector. Moreover, there is a need to revise the quality assessment practices both on a system level and on a facility level. How this should be done and organized is a topic that requires further study [35].

## Supplementary Information

**Additional file 1.** The survey“Educational opportunities for health care managers in Ukraine” (extract)

## Data Availability

The datasets used and/or analysed during the current study available from the corresponding author on reasonable request. All statistical methods were carried out in accordance with relevant guidelines and regulations.

## Abbreviations

BIHSENA: Bridging innovations, health and societies
CPD: Continuing professional development
GP: General practitioner
MED: Ukrainian-swiss project “Medical Education Development”
NHSU: The national health service of Ukraine
