# Supplementary material for: The perception of health care quality by primary health care managers in Ukraine
Source: BMC Health Serv Res. 2022 Jul 10;22:895. doi: 10.1186/s12913-022-08300-y (PMC9271244; doi:10.1186/s12913-022-08300-y)
Supplement: Supplementary file 1 — Additional file 1. The survey“Educational opportunities for health care managers in Ukraine” (extract) [file 12913_2022_8300_MOESM1_ESM.docx]

***Appendix A. The survey “Educational opportunities for health care managers in Ukraine” (extract)***

Dear health care managers,

We ask managers of the medical facilities, centers, outpatient clinics, feldsher-midwife stations as well as doctors who are in the reserve list for managerial positions to fill in the online survey “Educational opportunities for health care managers in Ukraine”.

In 2018, the Swiss Tropical and Public Health Institute (Swiss TPH) under the funding of the Swiss Agency for Development and Cooperation (SDC) launched the Medical Education Development project in Ukraine (MED).

The project covers all levels of medical education - undergraduate, graduate (internatura), post-graduate and continuous professional development - for family doctors and chiefs of primary health care facilities.

Since we are also developing educational products for health care managers, we would appreciate if you fill in the questionnaire on your experience and expectations. We need to understand:

- What are the sources you receive your knowledge and skills from? How interesting these sources are for you? What would you like to change in educational products you use now? What forms and regimen of studying is comfortable for you?
- What kinds of internal education you have in your health care facilities? How important they are for you? What would you change?
- What skills you have at the moment? What skills you feel you lack?

We ask you to fill in this survey which will take about 25 minutes from your side.

Within the project we will create educational modules for managers in health care. We will invite those who participated in this survey to take part in one of the planned online seminars.

Thank you for your time and answers.

**3. The block “Quality management in health care”**

3.1. What does the term ‘quality in health care’ mean to you? __________________________

3.2. Do you have quality management system in your health care facility?

Yes

No

3.3. If you have quality management system in your health care facility, please, describe how you assess quality. ____________________________________________________________

**5. The block “Information about survey participant”**

5.1. How old are you? _________________________________________________________

5.2. Indicate your post in health care facility ________________________________________

5.3. Are you in in the “reserve list” for management position at the moment?

Yes

No

5.4. Years of medical experience _________________________________________________

5.5. Years of management experience _____________________________________________

5.6. To what level does your facility belong to:

Primary

Secondary

Tertiary
